# Distinct Prognostic Values of Phospholipase C Beta Family Members for Non-Small Cell Lung Carcinoma

**DOI:** 10.1155/2019/4256524

**Published:** 2019-04-07

**Authors:** Tengfang Zhang, Xiaowei Song, Xiwen Liao, Xiangkun Wang, Guangzhi Zhu, Chengkun Yang, Xiaoyong Xie

**Affiliations:** ^1^Department of Cardiothoracic Surgery, The First Affiliated Hospital of Guangxi Medical University, Nanning, Guangxi Province 530021, China; ^2^Department of Gastrointestinal Glands, The First Affiliated Hospital of Guangxi Medical University, Nanning, Guangxi Province 530021, China; ^3^Department of Hepatobiliary Surgery, The First Affiliated Hospital of Guangxi Medical University, Nanning, Guangxi Province 530021, China

## Abstract

**Background:**

Non-small cell lung cancer (NSCLC) is a main cause of cancer-related mortality worldwide. The relationships of the phospholipase C beta (PLCB) enzymes, which are encoded by the genes PLCB1, PLCB2, PLCB3, and PLCB4, with NSCLC have not been investigated. Therefore, the aim of the present study was to identify any correlations between NSCLC prognosis and the expression patterns of PLCB family members.

**Materials and Methods:**

The prognostic values of the PLCB gene family members in NSCLC patients were evaluated using the “Kaplan–Meier plotter” database, which includes updated gene expression data and survival information of a total of 1,926 NSCLC patients. The GeneMANIA plugin of Cytoscape software was used to evaluate the relationships of the four PLCB family members at the gene and protein levels. Gene ontology enrichment analysis and KEGG pathway analysis were performed using the Database for Annotation, Visualization, and Integrated Discovery.

**Results:**

High mRNA expression levels of PLCB1, PLCB2, and PLCB3 were significantly associated with poor overall survival (OS) of all NSCLC patients and significantly associated with poor prognosis of adenocarcinoma. In contrast, high mRNA expression of PLCB4 was associated with better OS of adenocarcinoma patients. In addition, the expression levels of the PLCB family members were correlated to smoking status, clinical stage, and patient sex but not radiotherapy and chemotherapy outcomes.

**Conclusions:**

PLCB1, PLCB2, PLCB3, and PLCB4 appear to be potential biomarkers for the prognosis of patients with NSCLC. The prognostic values of the PLCB genes require further investigations.

## 1. Introduction

Lung cancer (LC) is the most frequently diagnosed cancer in both males and females (11.6% of all cases) and the primary cause of cancer-related mortality worldwide (18.4% of all cancer-related deaths). In addition, LC is the leading cause of cancer-associated mortality in men and the third most common in women [1]. In general, malignant lung tumors are classified as either small or non-small cell lung carcinoma (SCLC and NSCLC, resp.) [2], with the latter accounting for about 85% of all cases. Histologically, NSCLC is typically classified as one of three subtypes: squamous cell carcinoma (SCC), adenocarcinoma (AC), or large cell carcinoma [3]. Despite the extensive advancements in treatment of LC made over the last several decades, the benefits of surgery, radiotherapy, and chemotherapy are limited, particularly for those with locally advanced or distant metastatic disease (stage III/IV). Moreover, the conventional treatment for LC is whole-body chemotherapy with cisplatin, but the efficacy of such regimens is also limited [4]. Although several biomarkers for the prognosis of LC have been reported, such as ELF3 [5], miRNA-135 [6], and miRNA-34 [7], the survival rate of LC patients remains unsatisfactory. Accordingly, further studies to elucidate the underlying mechanisms of the initiation and progression of LC, as well as the identification of other prognostic molecular markers, are of crucial significance.

The phospholipase C beta (PLCB) class of phospholipases comprises four isozymes (b1–b4) which are encoded by varied genes [8]. PLCB3 is expressed in diverse tissue types [9, 10], while the tissue distribution of the other PLCB isozymes is more limited [10]. Members of the PLCB gene family have been associated with several diseases, including cancers, but any potential relationship with NSCLC remains unclear. PLCB1 is highly expressed by neuronal tissue [11], PLCB2 by hematopoietic cells [12], and PLCB4 by specific brain areas, which has no apparent involvement in graphical perception [13]. A previous study [14] reported that PLCB is a pivotal mediator of the progression of both SCLC and NSCLC. In addition, the augmented expression and the particular activation of PLCB1 by neuropeptide agonists suggest that PLCB1 is likely to play a substantial role in the stimulation of neuroendocrine growth factors that promote the progression of SCLC. However, no study has yet to investigate the expression patterns of PLCB isozymes in various subtypes of LC. Therefore, the primarily aim of the present study was to evaluate the prognostic values of the PLCB family members to provide new individualized treatment strategies and better prognostic indicators for NSCLC patients.

## 2. Materials and Methods

### 2.1. Kaplan–Meier Plotter (KM-Plotter)

The KM-plotter is an online survival analysis instrument designed to immediately assess the impact of genes on the prognosis of cancers of the breast, lung, ovary, liver, and stomach, with the use of genome-wide microarrays. Accordingly, the KM-plotter instrument was used in the present study to assess the prognostic values of PLCB genes in patients with NSCLC.

### 2.2. Bioinformatic Analysis

Analyses of Gene Ontology (GO) and Kyoto Encyclopedia of Genes and Genomes (KEGG) enrichment of PLCB genes were conducted using the Database for Annotation, Visualization, and Integrated Discovery (DAVID; v.6.8; https://david.ncifcrf.gov/home.jsp; accessed on August 8, 2018) [15]. Gene-gene and protein-protein interaction networks were investigated using the Gene Multiple Association Network Integration Algorithm (GeneMANIA: https://www.genemania.org/; accessed on August 14, 2018. STRING: https://string-db.org/) [16, 17].

### 2.3. Data Sources

In total, 1,926 patient samples were classified in accordance with the median and overall survival (OS) rates. Medical data, which included the patient's sex, smoking history, histological grade, American Joint Committee on Cancer stage, and success of surgery, radiotherapy, and applied chemotherapy for NSCLC, were collected from the Gene Expression Omnibus database (https://www.ncbi.nlm.nih.gov/geo/; accessed on August 5, 2018).

### 2.4. Survival Analysis of PLCB Family Members

A database was created with the use of the KM-plotter (http://kmplot.com/; accessed on September 5, 2018) to identify correlations between PLCB family members at the mRNA level and the prognosis and OS of NSCLC patients [18].

## 3. Results

### 3.1. Collection of Patient Data

In the current research work, the KM-plotter was employed to analyze the medical records of 1926 LC patients. Approval by the Ethics Committee was not required because no human participants or animals were involved in this study. And we collect basic clinical information on squamous cell carcinoma and adenocarcinoma in lung cancer (Table 1).

### 3.2. Interaction Analysis of PLCB Family Members at the Gene and Protein Levels

**Figure 1:**
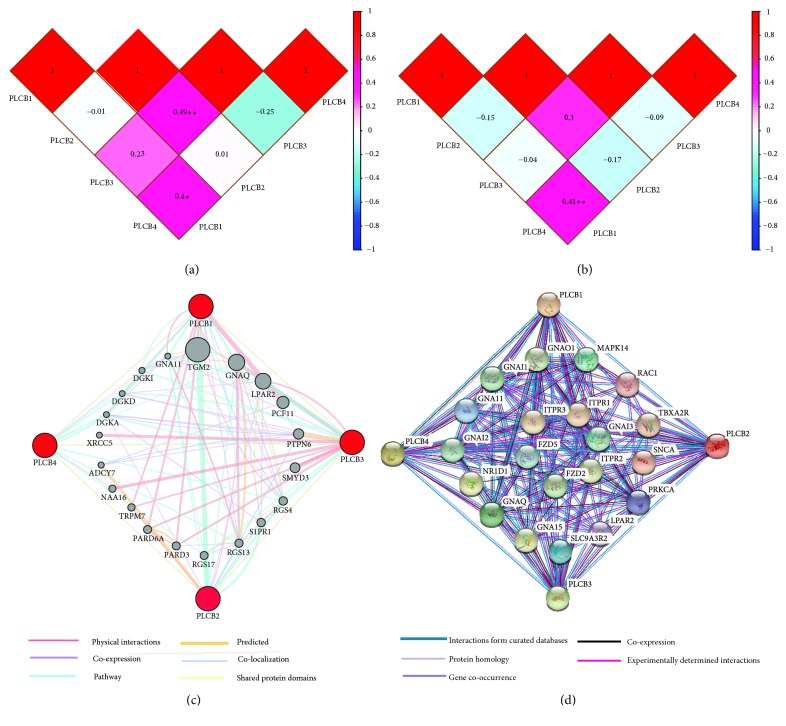
Interaction analysis of PLCB family members. (a) Pearson correlation of PLCB gene family members in lung adenocarcinoma. (b) Pearson correlation of PLCB gene family members in lung squamous cell cancer. (c) Gene-gene interaction network among PLCB gene family members. (d) Protein-protein interaction network among PLCB gene family members. Notes: PLCB, phospholipase C beta.

Pearson's correlation analysis was carried out with the use of the expression data of PLCB family members gathered from the Metabolic gEne RApid Visualizer (MERAV) gene expression database (http://merav.wi.mit.edu./, accessed on August 14, 2018). In lung AC, PLCB3 was significantly associated with PLCB2 (r = 0.49,* p* < 0.001; Figure 1(a)), while PLCB4 was significantly associated with PLCB1 (r = 0.4,* p* < 0.01; Figure 1(a)). In lung SCC, PLCB4 was significantly associated with PLCB1 (r = 0.41,* p* < 0.001). Detailed results are presented in Figure 1(b).

GeneMANIA was used for the correlation analysis of PLCB family members at the gene level to shed light on the relationships among pathways, shared protein domains, colocalization, coexpression, and prediction (Figure 1(c)). As revealed by the protein-protein interaction networks from STRING analysis, the PLCB genes were found to be linked to one another in an intricate network (Figure 1(d)).

### 3.3. Enrichment Analysis of GO Terms and KEGG Pathways

**Figure 2:**
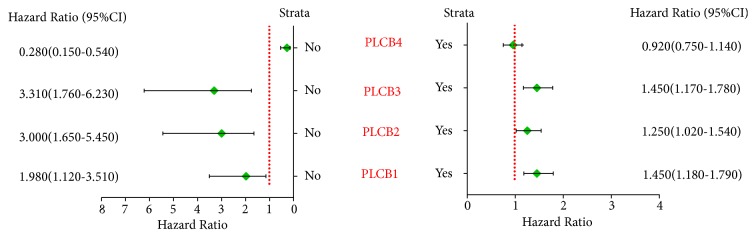
Correlation analysis between PLCB family members and smoking status. Notes: PLCB1, phospholipase C beta 1; PLCB2, phospholipase C beta 2; PLCB3, phospholipase C beta 3; PLCB4, phospholipase C beta 4; HR, hazard ratio; CI, confidence interval.

Correlation analysis among the PLCB gene family members and the five clinical factors of smoking status, clinical stage, radiotherapy outcome, chemotherapy outcome, and sex suggested that nonsmoking, but not smoking, status was significantly associated with PLCB4 (*p* = 4.4e−05 and 0.46, resp.), while PLCB1, PLCB2, and PLCB3 were significantly associated with both smoking and nonsmoking status (*p* = 0.00049, 0.017, 0.035, 0.00015, 0.00047, and 8.7e−05, resp.) (Figure 2).

**Figure 3:**
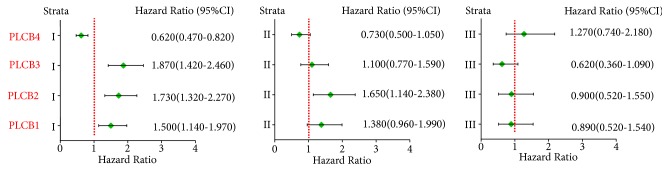
Correlation analysis between PLCB family members of clinical stage of NSCLC. Notes: PLCB1, phospholipase C beta 1; PLCB2, phospholipase C beta 2; PLCB3, phospholipase C beta 3; PLCB4, phospholipase C beta 4; HR, hazard ratio; CI, confidence interval.

**Figure 4:**
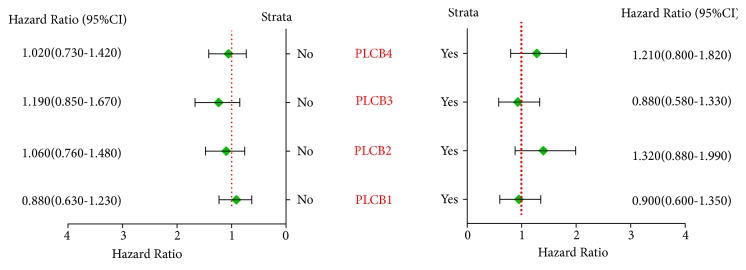
Correlation analysis between PLCB family members and chemotherapy outcomes of NSCLC. Notes: PLCB1, phospholipase C beta 1; PLCB2, phospholipase C beta 2; PLCB3, phospholipase C beta 3; PLCB4, phospholipase C beta 4; HR, hazard ratio; CI, confidence interval.

**Figure 5:**
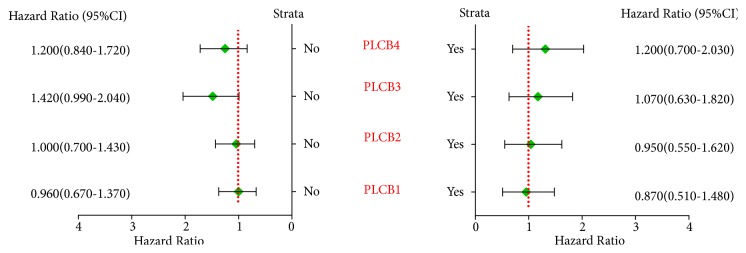
Correlation analysis between PLCB family members and radiotherapy outcomes of NSCLC. Notes: PLCB1, phospholipase C beta 1; PLCB2, phospholipase C beta 2; PLCB3, phospholipase C beta 3; PLCB4, phospholipase C beta 4; HR, hazard ratio; CI, confidence interval.

**Figure 6:**
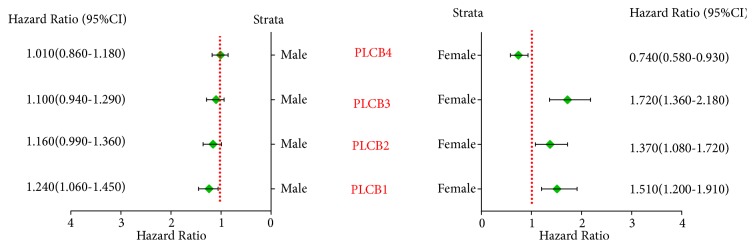
Correlation analysis between PLCB family members and gender of NSCLC. Notes: PLCB1, phospholipase C beta 1; PLCB2, phospholipase C beta 2; PLCB3, phospholipase C beta 3; PLCB4, phospholipase C beta 4; HR, hazard ratio; CI, confidence interval.

As revealed by the correlation analysis of PLCB family members with the clinical stage, grade I disease was significantly correlated with PLCB1, PLCB2, PLCB3, and PLCB4 (*p* = 0.0032, 6.6e−05, 6.4e−06 and 0.00059, resp.), while grade II disease was significantly correlated with PLCB2 (*p* = 0.0072) but not PLCB1, PLCB3, or PLCB4 (*p* = 0.085, 0.6, and 0.084, resp.). There was no correlation between grade III disease and PLCB1, PLCB2, PLCB3, and PLCB4 (*p* = 0.69, 0.71, 0.093, and 0.39, resp.; Figure 3). Further analysis suggested that there were no significant correlations between PLCB family members and radiotherapy and chemotherapy outcomes (all* p* > 0.05; Figures 4 and 5). Moreover, being female was significantly associated with PLCB1, PLCB2, PLCB3, and PLCB4 (*p* = 0.00047, 0.008, 5.1e−06, and 0.0095, resp.), while being male was significantly associated with PLCB1 (*p* = 0.0072) but not PLCB2, PLCB3, or PLCB4 (*p* = 0.065, 0.23, and 0.88, resp.; Figure 6)

GO analysis, with the terms of “biological process,” “cellular component,” and “molecular function” as well as KEGG pathway enrichment analysis, was conducted using DAVID. The top five results obtained from the enrichment analysis included phosphatidylinositol phospholipase C activity, together with inositol phosphate metabolism, signal transducer activity, lipid catabolism, and phospholipase C activity (Table 2). The top five results of the enriched KEGG pathways included inositol phosphate metabolism, gap junction, the Wnt signaling pathway, the calcium signaling pathway, and the chemokine signaling pathway (Table 3).

### 3.4. Survival Curve Analysis of PLCB Family Members Using the KN-Plotter

**Figure 7:**
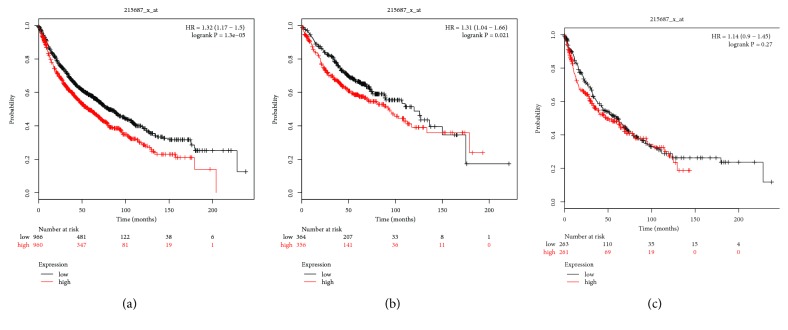
Survival analysis of PLCB1 (215687_x_at) in NSCLC. (a) Survival analysis of PLCB1 in both adenocarcinoma and squamous cell cancer. (b) Survival analysis of PLCB1 in adenocarcinoma. (c) Survival analysis of PLCB1 in squamous cell cancer. Notes: NSCLC, non-small cell lung carcinoma; PLCB1, phospholipase C beta 1; HR, hazard ratio.

Assessment of the prognostic values of PLCB family members was performed using the KM-plotter online website. The Affymetrix ID of PLCB1 is 215687_x_at. There were statistically significant correlations between the tissue type and AC, as well as the SCC type [*p* = 1.3e−05, hazard ratio (HR) = 1.32, 95% confidence interval (CI) = 1.17–1.5; Figure 7(a); p = 0.021, HR = 1.31, 95% CI = 1.04–1.66, Figure 7(b); p = 0.88, HR = 1.14, 95% CI = 0.9–1.45; Figure 7(c)].

**Figure 8:**
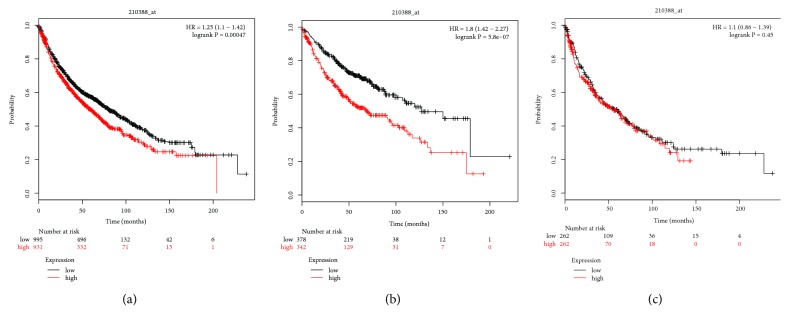
Survival analysis of PLCB2 (210388_at) in NSCLC. (a) Survival analysis of PLCB2 in both adenocarcinoma and squamous cell cancer. (b) Survival analysis of PLCB2 in adenocarcinoma. (c) Survival analysis of PLCB2 in squamous cell cancer. Notes: NSCLC, non-small cell lung carcinoma; PLCB2, phospholipase C beta 2; HR, hazard ratio.

Next, the prognostic value of PLCB2 mRNA expression was investigated using the KM-plotter. The Affymetrix ID of PLCB2 is 210388_x_at. High mRNA expression of PLCB2 was associated with a poor OS of every NSCLC patient (p = 0.00047, HR = 1.25, 95% CI = 1.1–1.42; Figure 8(a)), while elevated mRNA expression of PLCB2 was associated with the worse OS of AC patients (p = 5.8e−07, HR = 1.8, 95% CI = 1.42–2.27; Figure 8(b)), as well as SCC patients (p = 0.42, HR = 1.1, 95% CI = 0.86–1.39; Figure 8(c)).

**Figure 9:**
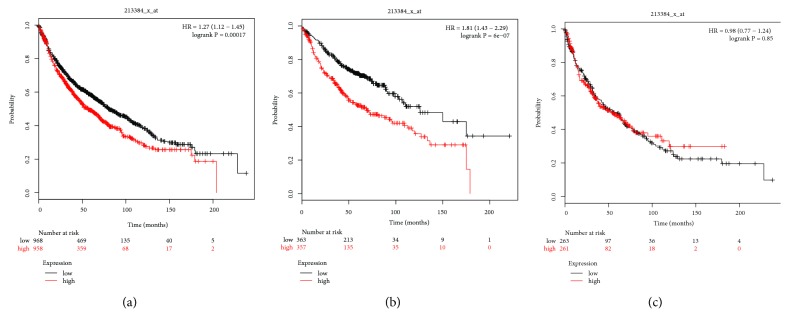
Survival analysis of PLCB3 (213384_x_at) in NSCLC. (a) Survival analysis of PLCB3 in both adenocarcinoma and squamous cell cancer. (b) Survival analysis of PLCB3 in adenocarcinoma. (c) Survival analysis of PLCB3 in squamous cell cancer. Notes: NSCLC, non-small cell lung carcinoma; PLCB3, phospholipase C beta 3; HR, hazard ratio.

The prognostic value of PLCB3 mRNA expression, as determined with the KM-plotter instrument, is described in Figure 3. The Affymetrix ID of PLCB3 is 213384_x_at. Elevated mRNA expression of PLCB3 was associated with the worse OS for every NSCLC patient (p = 0.00017, HR = 1.27, 95% CI = 1.12–1.45; Figure 9(a)) as well as AC patients (p = 6e−07, HR = 1.81, 95% CI = 1.43–2.29; Figure 9(b)). Nonetheless, the high mRNA expression of PLCB3 was significantly correlated with an improved OS of SCC patients (p = 0.85, HR = 0.98, 95% CI = 0.77–1.24; Figure 9(c)).

**Figure 10:**
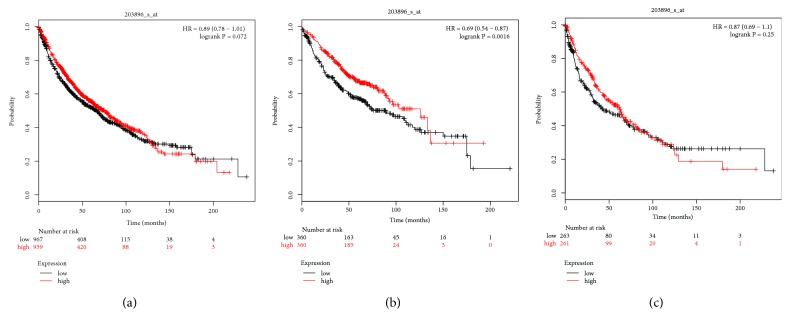
Survival analysis of PLCB4 (203896_s_at) in NSCLC. (a) Survival analysis of PLCB4 in both adenocarcinoma and squamous cell cancer. (b) Survival analysis of PLCB4 in adenocarcinoma. (c) Survival analysis of PLCB4 in squamous cell cancer. Notes: NSCLC, non-small cell lung carcinoma; PLCB4, phospholipase C beta 4; HR, hazard ratio.

The Affymetrix ID of PLCB4 is 203896_s_at. There was no significant correlation between AC and SCC and the histological type (p = 0.072, HR = 0.89, 95% CI = 0.78–1.01; Figure 10(a); p = 0.87, HR = 0.25, 95% CI = 0.69–1.1; Figure 10(c), resp.), while there was a significant correlation with AC (p = 0.0016, HR = 0.69, 95% CI = 0.54–0.87; Figure 10(b)).

## 4. Discussion

Some reports have suggested that the expression patterns of the PLCB isozymes are reduced or lost in human diseases. For instance, PLCB3 gene expression is lost in multiple endocrine neoplasia type 1 [19] and lowered in abnormal platelet aggregation [12]. Besides that, a recent study reported that the expression of catalytically inactive PLCB inhibits progression of SCLC, indicating that signaling through Gq and PLCB is a dominant pathway involved in the transformation and growth of cancer cells [20].

There are numerous reports linking changes in the mRNA expression of PLCB1 in NSCLC cell lines and/or tumor tissues [14]. However, there has been no study on the prognostic values of the mRNA expression profiles of the PLCB gene family members in NSCLC patients.

The KM-plotter is an extensively utilized database composed of gene expression data as well as survival information [21]. PLCBs are known to be activated by two G-protein families: Gi/o, which is inhibited by pertussis toxins (PTX), and Gq, which is resistant to PTX. PLCB1 and PLCB3 which are activated by the subunits of Gq, whereas PLCB2 is activated primarily by the *α* and *β* subunits of Gi/o [10, 22, 23]. As also reported, PLCB2 and PLCB3 are likely involved in the proliferation of Ewing's sarcoma cells [24]. PLCB3 is an isoform associated with the phospholipase C family that is correlated with membrane receptors by means of isoform-specific processes. In addition, PLCB3 has been observed in the small-diameter, presumptive nociceptor and the dorsal root ganglion neurons of rats [25]. PLCB3 constitutes the upstream target of the protein kinase C epsilon, which contributes to the inflammatory response.

Numerous studies have investigated the prognostic values of the PLCB gene family members in cancer patients. Upregulated expression of PLCB1 has been associated with the proliferation of tumor cells as well as in poor prognosis of hepatocellular carcinoma patients [26]. In addition, high quantities of this protein have been narrowly linked to a poor prognosis of patients with mammary carcinoma, suggesting that PLCB2 is likely to be involved with both the growth and deterioration of the malignant phenotype [27]. Moreover, PLCB4 has been associated with the catalysis of inositol 1, 4, 5-trisphosphate and diacylglycerol from phosphatidylinositol 4,5-bisphosphate in signal transduction, together with having a significant role in visual activities. Furthermore, a study on the significance of PLCB1 inactivation on the antiproliferative impacts of ET-18-OCH3 found that catalytically inactive PLCB1 hampers the clonogenic development of SCLC cells, as compared with NSCLC cells [20]. As revealed by the present study, PLCB1 expression is likely to have latent medical significance in SCLC, coupled with its function in regulating the development factor-mediated signaling. The SCLC neuroendocrine PLCB1 phenotype has diagnostic as well as prognostic value, together with being a target for therapeutic intervention [28]. Hence, PLCB1 expression is likely to be an exclusive characteristic of SCLC cells that could be exploited in the same manner. In addition, PLCB1 expression is significantly greater in SCLC than NSCLC; thus further investigations are required to clarify the function of PLCB1 in LC biology and to assess the value of PLCB1 as a target for treatment of this disease. The findings of the current study illustrate the prognostic value of PLCB members at the mRNA level in NSCLC. Moreover, elevated mRNA expression levels of PLCB1, PLCB2, and PLCB3 were linked to poor OS of every NSCLC patient and were significantly associated with poor prognosis of AC. In contrast, elevated mRNA expression of* PLCB4* was linked to improved OS of AC patients. In addition, the mRNA expression levels of the* PLCB* family members were associated with smoking status, as well as clinical stage and patient sex, but not with radiotherapy and chemotherapy outcomes.

Nicotine is known to regulate numerous biological roles, such as cell proliferation, in addition to invasion, inflammation, apoptosis, and angiogenesis [29]. By inducing the secretion of stem cell factors, nicotine has the ability to regulate the development and metastasis of NSCLC [30]. However, there is no evidence of a direct association between nicotine and PLCB expression in NSCLC. Nonetheless, numerous investigations have suggested that the Gq/11 family of G protein-coupled PLCB1 isozyme is likely to be involved in neuroadaptive mechanisms [31]. In the current report, nonsmoking status was significantly associated with PLCB4, while PLCB1, PLCB2, and PLCB3 were significantly associated with both smoking and nonsmoking status. PLCB4 was not significantly associated with smoking status. Nonetheless, there have been limited studies on the prognostic relevance of PLCB protein expression in NSCLC patients. Recent observations shed light on the fact that some PLCB family members are likely to have prognostic values in NSCLC patients, but additional investigations are required.

There were some limitations to the current research which should be addressed. First, the study cohort was relatively small; thus future studies with larger cohorts involving multiple centers with patients of various races are required to verify these findings. To address these issues, we are planning well-designed functional verification studies, which include both* in vitro* and* in vivo* models, in the near future. The discovery or application value of this article is still in its primary stage. Our study found that the PLCB family may be a factor influencing the prognosis of lung cancer. Monitoring this factor in advance may help to understand the patient's prognosis. This speculation also needs to be further validated in animal experiments and clinical trials. At present, genetic testing is a good and feasible method in clinical practice. In terms of feasibility, we also need the joint efforts of the government or those who have established rules and regulations, so that we can better apply scientific research results to clinical practice and better serve patients. As for the other potential shortcomings, PLCB1, PLCB2, PLCB3, and PLCB4 were all associated with the prognosis of NSCLC and smoking is likely to impact the expression of genes as well as the clinical stage of disease. Thus, PLCB1, PLCB2, PLCB3, and PLCB4 should be considered as potential prognostic biomarkers for NSCLC. Second, the data for 1926 LC patients in this paper were obtained from The Kaplan-Meier plotter (https://kmplot.com/; accessed on September 5, 2018), and data from 14 different platforms were collected. The characteristics of the samples collected in different data platforms were different. Among the sample characteristics, it did not collect them in detail in which nonsmoking LC patients should be divided into never smoking and ex-smoking patients. Also the information on the different chemotherapy methods and chemotherapy cycles in the sample characteristics was not collected. Therefore, we regret that we could not further explore the relationship between PLCB gene family members and patients who never smoked or ex-smoked and we have not been able to further analyze the relationship between PLCB gene family members and different chemotherapy and also failed to analyze the relationship between PLCB gene family members and the chemotherapy cycle relationship. In spite of these limitations, to the best of our knowledge, the current study is the first to explore the correlations between the mRNA expression patterns of PLCB family members to predict the OS of patients with NSCLC. Besides, these findings bring an understanding of the role of PLCB genes in the clinical outcomes of cancer and further illustrate the application for patient prognosis as well as decision-making in NSCLC management.

## 5. Conclusions

In conclusion, the aim of the present study was to explore the associations between NSCLC prognosis and the expression patterns of PLCB gene family members. The results showed that high mRNA expression levels of PLCB1, PLCB2, and PLCB3 were linked to a poor OS of every NSCLC patient and were significantly associated with poor prognosis of AC. In contrast, elevated mRNA expression of PLCB4 was associated with better OS of AC patients. Additionally, the expression levels of the PLCB family members were correlated to smoking status, as well as clinical stage and patient sex, but not radiotherapy and chemotherapy outcomes. Accordingly, the PLCB family is likely to have prognostic value in NSCLC patients, but additional investigations are needed.

## Figures and Tables

**Table 1:** Demographic and clinical data for 1926 LC patients.

	adenocarcinoma	squamous cell carcinoma	t/F	P
*Gender*			49.116	**<0.001**
male	344	342		
female	318	129		
*Smoking history*			93.527	**<0.001**
smoked	246	244		
never smoked	143	9		
*Chemotherapy*			0.999	0.318
Yes	36	42		
No	21	35		
*AJCC stage T*			31.786	**<0.001**
1	123	106		
2	105	160		
3	4	25		
4	0	13		
*AJCC stage N*			12.375	**0.002 **
0	184	208		
1	44	77		
2	3	19		
*AJCC stage M*			0.129	0.720
0	231	300		
1	1	2		
*Grade*			-	-
I	0	22		
II	0	93		
III	0	15		
*Stage*			32.910	**<0.001**
I	370	172		
II	136	100		
III	24	43		
IV	4	0		

Notes: ^&^gender information is unavailable in 793 patients. ^&^Smoking history information is unavailable in 1284 patients. ^&^Chemotherapyinformation is unavailable in 1792 patients. ^&^AJCC stage T, N, and M information is unavailable, respectively, in 1390, 1391, and 1392 patients. ^&^Grade information is unavailable in 1796 patients. ^&^Stage information is unavailable in 1077 patients.

Notes: AJCC, American Joint Committee on Cancer.

**Table 2:** Enrichment analysis of gene ontology of PLCB family members.

Category	Term	Count	%	P value	FDR
GOTERM_MF_DIRECT	GO:0004435~phosphatidylinositol phospholipase C activity	4	0.575539568	**3.64888E-09**	2.34757E-06
GOTERM_BP_DIRECT	GO:0043647~inositol phosphate metabolic process	4	0.575539568	**2.05513E-08**	1.86892E-05
GOTERM_BP_DIRECT	GO:0016042~lipid catabolic process	4	0.575539568	**1.25183E-07**	0.000113841
GOTERM_MF_DIRECT	GO:0004629~phospholipase C activity	3	0.431654676	**1.64167E-06**	0.001056194
GOTERM_MF_DIRECT	GO:0004871~signal transducer activity	4	0.575539568	**1.73924E-06**	0.001118967
GOTERM_BP_DIRECT	GO:0035556~intracellular signal transduction	4	0.575539568	**1.37229E-05**	0.012478834
GOTERM_BP_DIRECT	GO:0007223~Wnt signaling pathway, calcium modulating pathway	3	0.431654676	**1.57453E-05**	0.014317811
GOTERM_MF_DIRECT	GO:0005509~calcium ion binding	4	0.575539568	**7.63169E-05**	0.049089644
GOTERM_CC_DIRECT	GO:0005829~cytosol	4	0.575539568	**0.006014477**	4.193516337
GOTERM_MF_DIRECT	GO:0005516~calmodulin binding	2	0.287769784	**0.033215354**	19.53326842

Notes: PLCB, phospholipase C beta; FDR, false discovery rate.

**Table 3:** Enrichment analysis of KEGG pathways of PLCB family members.

Term	Count	%	P value	FDR	Genes
hsa00562:Inositol phosphate metabolism	4	0.575539568	**1.04E-06**	9.62E-04	PLCB3, PLCB4, PLCB1, PLCB2
hsa04540:Gap junction	4	0.575539568	**2.00E-06**	0.00184768	PLCB3, PLCB4, PLCB1, PLCB2
hsa04310:Wnt signaling pathway	4	0.575539568	**7.80E-06**	0.007215304	PLCB3, PLCB4, PLCB1, PLCB2
hsa04020:Calcium signaling pathway	4	0.575539568	**1.71E-05**	0.015825071	PLCB3, PLCB4, PLCB1, PLCB2
hsa04062:Chemokine signaling pathway	4	0.575539568	**1.92E-05**	0.017766346	PLCB3, PLCB4, PLCB1, PLCB2
hsa04015:Rap1 signaling pathway	4	0.575539568	**2.77E-05**	0.025615883	PLCB3, PLCB4, PLCB1, PLCB2
hsa05200:Pathways in cancer	4	0.575539568	**1.83E-04**	0.168911037	PLCB3, PLCB4, PLCB1, PLCB2

Notes: PLCB2, phospholipase C beta 2; PLCB3, phospholipase C beta 3; PLCB4, phospholipase C beta 4; FDR, false discovery rate.

## Data Availability

The data used to support the findings of this study are included within the article.
